# Take patients seriously when they say financial incentives help with adherence

**DOI:** 10.1192/bjb.2022.76

**Published:** 2023-06

**Authors:** Nathan Hodson, Madiha Majid, Ivo Vlaev, Swaran Preet Singh

**Affiliations:** 1 Warwick Medical School, UK; 2 Warwick Business School, UK

**Keywords:** Ethics, qualitative research, service users, stigma and discrimination, consent and capacity

## Abstract

Small financial incentives have been proven effective at promoting healthy behaviours across medicine, including in psychiatry. There are a range of philosophical and practical objections to financial incentives. Drawing on the existing literature, specifically attempts to use financial incentives to promote antipsychotic adherence, we propose a ‘patient-centred’ view of evaluating financial incentive regimes. We argue that there is evidence that mental health patients like financial incentives, considering them fair and respectful. The enthusiasm of mental health patients for financial incentives lends support to their use, although it does not invalidate all objections against them.

The idea of giving patients small financial incentives for accepting treatment elicits strong reactions from mental health staff.^1^ It can make professionals uncomfortable, perhaps because of an acute awareness of the risk of replaying historical abuses of mental health patients. It is right to interrogate novel practices, but a central part of that analysis should be listening to the perspectives of the patients themselves. Contrary to the notion that financial incentives are problematic, we will argue that the best evidence shows that mental health patients find financial incentives helpful, respectful and fair, and that professionals should take these views seriously.

## Financial incentives in mental health

Financial incentives have been implemented in diverse settings across healthcare, proving effective at promoting smoking cessation, anti-retroviral therapy adherence and vaccination, among other healthy behaviours.^2^ In psychiatry, financial incentives have been shown to improve adherence to antidepressants and long-acting injectable antipsychotics, as well as reducing substance use.^3–5^ In the UK, the new Office for Health Improvement and Disparities has a remit including public mental health and financial incentives, but has yet to combine the two, and similar programmes are emerging elsewhere in the world.^6^ It seems likely that mental health policy around the world will increasingly grapple with the appropriateness of financial incentives.

The important distinction between ‘paying’ and ‘incentivising’ can easily be lost. Employees are paid to do a job for someone else. In financial incentive arrangements, by contrast, patients are incentivised to do something primarily for their own benefit. Far from threatening staff expertise, incentives can be construed as an additional string to the bow of services, along with motivational interviewing, home visits and community treatment orders, to help patients engage with collaboratively developed treatment plans and protect their mental health.

Against this background, rehearsing the potential objections to financial incentives is worthwhile. Financial incentives have been described as ‘coercive’, ‘exploitative’ and an affront to dignity. Others have objected that it is the wrong way to spend finite health service resources, suggesting patients might use the money to buy drugs that exacerbate their symptoms. Financial incentives might theoretically reduce patients’ intrinsic motivation to participate in mental healthcare. Offering incentives to those with low adherence could result in patients skipping doses so that they are offered a financial incentive, or could damage the therapeutic relationship between services and those with good adherence. Some people might even fear that professionals’ skills building trust and relationships with patients risk being devalued. There are also legitimate safeguarding concerns and practical challenges to overcome when giving vulnerable people monthly cash bonuses and, at some point, withdrawing them.^3^ A comprehensive review of objections to financial incentives, and whether these have empirical support, is available elsewhere.^3^

It would be easy to conclude that these objections – ranging from philosophical to pragmatic – fatally undermine the project. But we propose a different place to begin: the patient. When we begin with the experiences and perspectives of patients who have had their treatment supported by financial incentives, the picture is much more positive. From this perspective, financial incentives may be acceptable and appropriate, objections less clear cut. In the next section we will support this view with evidence from the large body of research into using financial incentives to promote antipsychotic adherence.

## Patient perspectives

First, survey data shows that mental health patients like financial incentive regimes and find them acceptable. After 12 months being offered financial incentives for adherence to antipsychotic depot treatment, 68% of patients reported they thought financial incentives were a good idea.^7^ Only 47% of staff were convinced, reflecting a mismatch between patient enthusiasm and staff wariness. Even though they were not asked about it directly, 41% of participants in this study spontaneously reported that they liked the financial incentive because it meant they ended up with more money. Remarkably, only 6% of staff identified this advantage and, given it was not in the interview regime, it seems it had not occurred to the researchers either. There's clearly a mismatch between cautious clinicians and their patients – and rightly so. But for the mental health patients getting it, it is easy to see the attraction of a small cash bonus every month. Even though depot injections provide a stressful, painful reminder of their mental illness, one patient pithily put it this way: ‘money makes it better’.^8^

Before offering financial incentives to patients, some staff expressed concerns about the fairness of offering incentives, fearing that it wrongly puts pressure on patients, thereby coercing them to take medication. But patients who have received financial incentives see the world in a more straightforward way: 76% endorse the statement that it is good to reward good behaviour, whereas only 36% of staff saw it that way.^7^ This difference is telling, and helps us see the situation through the eyes of patients. These statements suggest that many mental health patients don't appear to view the choice between adherence and non-adherence as selecting between finely balanced options where a small incentive can push them to act against their preferences. Nor do they perceive incentives as overwhelming their will or coercing them with an offer that is ‘too good to refuse’. Rather, they perceive adherence as a self-control challenge to overcome. With this framing, success leads to a sense of accomplishment for which a reward is by no means incongruous.

Some doctors have feared that their relationships with patients could be harmed by imposing on them a financial incentive regime perceived as a manipulation. Shaw has argued that using financial incentives in psychiatry undermines patient dignity and privacy and could threaten the therapeutic alliance.^9^ But patients are more adaptive than that view gives them credit for and generally take financial incentives in their stride. After a year of financial incentives, 84% of patients denied that their therapeutic relationships had worsened – and staff reported the same thing.^7^ In fact, in one study 73% of mental health staff reported better relationships with patients: patients became more trusting and even started actively phoning up for help resolving barriers to attendance.^10^ Strikingly, two studies found that around 20% of participants made jokes about wishing they could have their depot earlier than scheduled – a level of ‘banter’ indicating warm, relaxed *bonhomie* and a far cry from the resentful reluctance one might expect if patients begrudged the incentive regime.^8,10^

Finally, some staff have suggested that financial incentives should be replaced with tokens for therapeutic activities such as sport.^11^ Aside from the fact that this approach would entail deliberately withholding activities identified as therapeutic from certain (disorganised, mistrustful) patients, mental health patients have not expressed any interest in non-cash incentives. Even patients’ mothers endorse using cash incentives rather than vouchers.^8^ In follow-up interviews, many patients have suggested that larger cash incentives would have been better (although their mothers insisted incentives worth around £10–20 were just right).^8^ Our recent systematic review found no evidence of patients feeling that the incentives were so large as to create problems.^3^

## Preferences and policy

In several domains the evidence has shown differences between staff and patient experiences of financial incentives. Staff often appear not to expect (and not to notice) how patients feel about financial incentives. The existing evidence shows that patients with major mental illness like financial incentives and are generally much more relaxed about the arrangement than doctors. These differences should prompt psychiatrists to re-evaluate any objections.

Clearly, the stated preferences of patients cannot be the only criterion for healthcare service design. The first priority of doctors should be health and there are many situations where, owing to behavioural biases or information asymmetry, patients express preferences for interventions that are bad for their health; doctors are under no obligation to cater to patient preferences for their own sake. Indeed, doctors, commissioners and policymakers also have a duty to consider the implications of any novel intervention in order to avoid harming patients, so it is right for professionals to think carefully about the potential ethical objections listed above, just as it is incumbent on healthcare leaders to evaluate cost-effectiveness and consider ways of overcoming practical barriers to implementation. But in light of this evidence of the patient experience, critics should consider whether they may have overlooked arguments in favour of financial incentives that are easier to recognise from the perspective of the patient.

Our intention is not to criticise those with concerns about financial incentives. The mismatch between staff perspectives and patient perspectives reveals the need for more work to build a consensus among staff in support of financial incentives. Beyond identifying that patients find financial incentives acceptable, advocates of financial incentives should seek to understand and address any other concerns held by staff.

We have argued that patients are enthusiastic about financial incentives, finding them respectful and fair. Any objections should be considered in light of this empirical evidence in order to move the debate forward.

## Data Availability

Data availability is not applicable to this article as no new data were created or analysed in this study.

## About the authors

**Nathan Hodson** is a National Institute for Health and Care Research (NIHR) Academic Clinical Fellow in the Division of Mental Health and Wellbeing at Warwick Medical School, University of Warwick, UK. **Madiha Majid** is an NIHR Academic Clinical Fellow in the Division of Mental Health and Wellbeing at Warwick Medical School, University of Warwick, UK. **Ivo Vlaev** is Professor of Behavioural Science at Warwick Business School, University of Warwick, UK. **Swaran Preet Singh** is Professor of Social and Community Psychiatry in the Division of Health Sciences at Warwick Medical School, University of Warwick, UK.
